# Feature fusion-enhanced t-SNE image atlas for geophysical features discovery

**DOI:** 10.1038/s41598-025-01333-3

**Published:** 2025-05-17

**Authors:** Leonardo Portes, Guillaume Pirot, Michel M. Nzikou, Jeremie Giraud, Mark Lindsay, Mark Jessell, Edward Cripps

**Affiliations:** 1 Department of Mathematics and Statistics, The University of Western Australia, 35 Stirling Highway, Crawley, 6009 Australia; 2 ARC Centre for Data Analytics for Resources and Environments (DARE), Perth and Sydney, Australia; 3 Centre for Exploration Targeting, School of Earth and Oceans, The University of Western Australia, 35 Stirling Highway, Crawley, 6009 Australia; 4 GeoResources, Université de Lorraine, CNRS, Nancy, F-54000 France; 5 Commonwealth Scientific and Industrial Research Organization, Mineral Resources, 26 Dick Perry Ave, Kensington, WA 6151 Australia

**Keywords:** Geology, Geophysics

## Abstract

The discovery and identification of geophysical features from diverse gridded datasets play a pivotal role in understanding geological phenomena. Traditional tools tailored to identify specific signatures, such as lineaments in magnetic data, do not account for (1) the *naturally* occurring complexity of the causative geology which results from competing and overprinting processes and (2) often overlook important related patterns present in other forms of data (e.g., gradients in the associated gravity data). To address those limitations, we propose a two-step data-driven approach that integrates diverse gridded datasets and autonomously reveals inherent patterns without predefined assumptions in terms of feature geometry. Utilizing Haralick texture descriptors, we encode data patches from different geophysical datasets as points in a high-dimensional unified representation space, facilitating seamless data fusion. This representation is then nonlinearly projected into a two-dimensional space using t-distributed stochastic neighbor embedding (t-SNE), forming an interactive “t-SNE Atlas”. This atlas positions tiles from each dataset according to their position in the t-SNE while linking them back to their original geographical coordinates. Such a setup allows earth scientists to intuitively navigate through the data, exploring complex relationships between geophysical responses and geological structures, thus facilitating the discovery of new insights, the formulation of hypotheses, and the exploration of non-trivial connections. This is illustrated using magnetic and gravity data covering $58,800 {\textrm{km}}^2$ in the east Yilgarn Craton, Western Australia. Our methodology is adaptable and can be extended to include various other gridded datasets like gamma-ray spectrometry data, satellite imagery, and digital terrain models, thus broadening its applicability and enhancing geoscientific exploration. Our approach not only reveals subtle geophysical patterns but also offers practical benefits for exploration geologists. The atlas enables the identification of geological settings associated with mineral prospectivity and can serve as a pre-planning tool for pinpointing promising exploration sites, potentially accelerating discovery timelines.

## Introduction

Since the early days analysing digital images, researchers have tried to automatically extract meaning from them, both by enhancing the images so that the information is more readily understood by the human eye and brain and by the automatic extraction of features from the images themselves^1,2^. With the release of the first Landsat earthward looking satellite image of the Dallas Fort Worth area taken on July 25, 1972, earth scientists started to use digital data to interpret near-surface geology, and all of the interpretive and automated tools developed over the previous decade were applied to these multi-band images^3–5^. In 1978 Huntington and Green, research scientists at the Commonwealth Scientific and Industrial Research Organisation (CSIRO) applied the software developed process and analyse Landsat data to airborne geophysical data, and since that time there has been a steady stream of researchers focused on extracting meaning from these gridded geophysical datasets in plan view^6^ and section view^7–10^.

One of the drawbacks of using feature extraction methods that search for *specific* shapes (e.g., lineaments) in natural scenes such as geophysical data is that geological datasets are complex and do not follow simple rules, as opposed to, e.g., face recognition. Indeed, human faces have a consistent and well-defined structure, with most faces having common features in predictable locations (e.g., two eyes symmetrically located above one nose). This implies on a low-dimensional feature space with a simple structure, which enables the use of simple techniques like principal components analysis (PCA) to capture the most significant features with fewer components^11^. In contrast, geological datasets are high-dimensional, exhibit irregular patterns with complex geophysical signatures, and lack the same level of predictability and consistency, making feature extraction a (much) more challenging task^12–14^. Extracting meaningful features from such data often requires sophisticated methods to manage the higher dimensionality and complexity of the feature space itself^15–23^.

**Fig. 1:**
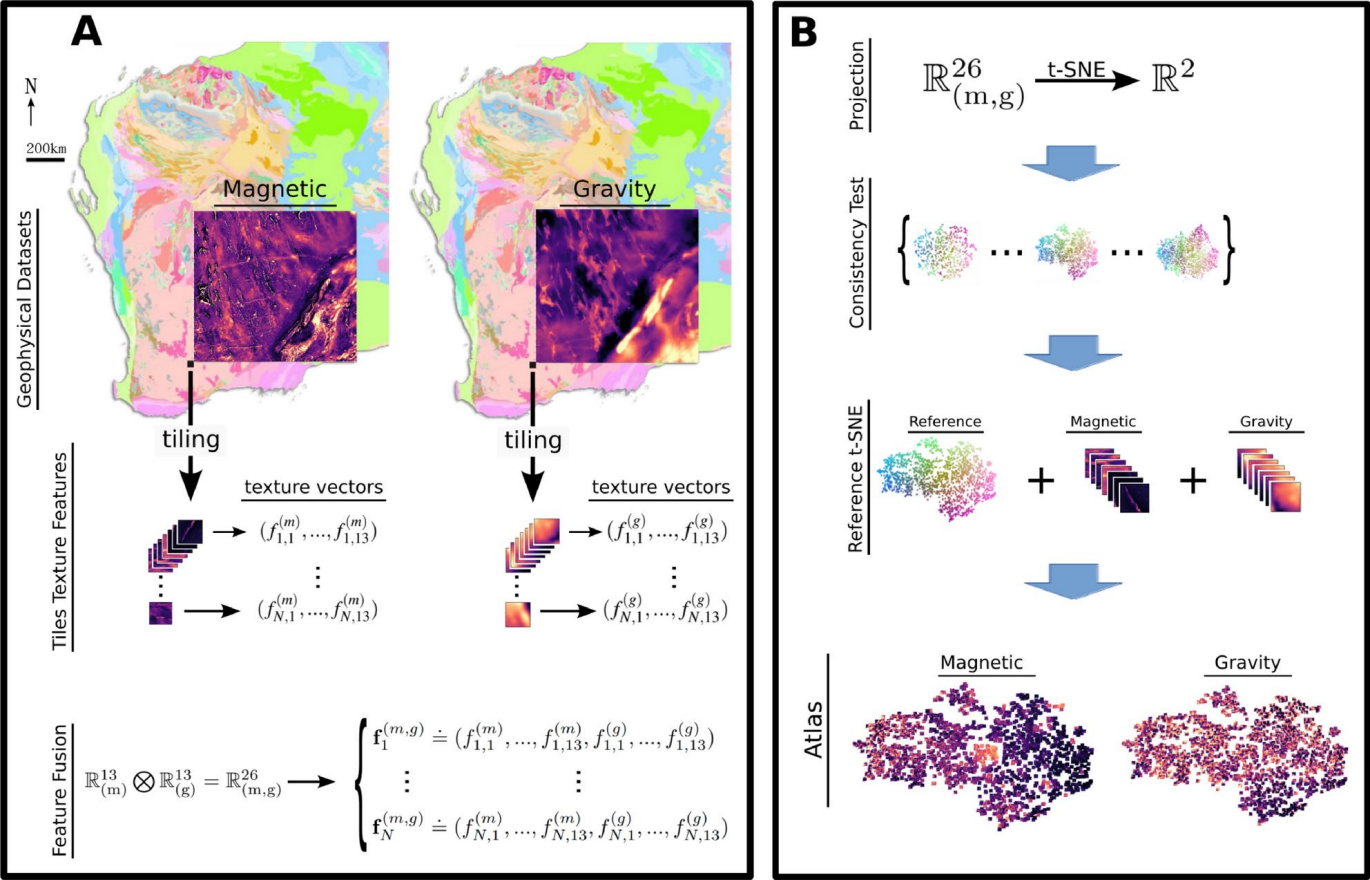
Schematic diagram of the workflow. (**A**) Different geophysical datasets (magnetic and gravity) from the same study area^32,33^ are segmented into small tiles, which are then encoded as points based on their Haralick texture descriptors (vectors) within two 13-dimensional spaces. A unified 26-dimensional feature space is then obtained by concatenating these two feature vectors, achieving feature fusion for subsequent analysis. (**B**) The high-dimensional feature space is nonlinearly projected into two dimensions using t-SNE, with multiple embeddings generated across different perplexity values. From these candidates, a reference t-SNE projection is selected based on consistency in clustering formation and the desired balance between preserving the local *versus* global structure. Subsequently, two maps (magnetic and gravity) are created by positioning image tiles according to their coordinates in this reference t-SNE projection. Gravity and magnetic images shown with the background map from GSWA 2020^34^.

In this study, we propose a hybrid method that attempts to cluster *naturally* occurring geophysical features within small image patches and provide these clusters in a manner that allows the earth scientist to interpret these clustered images using their prior knowledge of the region. The workflow is summarized in Fig. 1. Specifically, we explore the use of Haralick texture descriptors^24–27^ to encode the (high-dimensional) patches and integrate diverse geophysical data into a cohesive lower dimensional space, and the t-distributed stochastic neighbor embedding (t-SNE)^28–31^ for the nonlinear projection in 2D to build a *t-SNE atlas* for organizing multimodal geophysical information in a sensible way (see Methods for a detailed description). We also enhance this tool with interactive features such as linking the t-SNE output to the *geographical locations* of the original data and advocate its use as a dynamic tool for geophysical exploration, allowing a subject matter expert to “navigate” large datasets in search of insights and meaning. The interactive tool is available at https://www.loopwms.xyz/wagravmag/. As one intermediary step, we also suggest a *perplexity consistency* test to select an optimal 2D embedding from a set of t-SNEs, thereby supporting more accurate interpretations and analyses of the data (see Methods). Our approach is illustrated with magnetic and gravity data spanning $58,800 \textrm{km}^2$ from the east Yilgarn Craton region in Western Australia. This is a geologically complex area, and a focal point for mineral exploration, attracting considerable research and exploration efforts. The main novelties and advantages of our method are the ability to integrate diverse geophysical information (i.e., data fusion through Haralick texture descriptors), its visualization and map navigation capabilities (i.e., 2D t-SNE projection, interactivity and link to geophysical position), not too be limited by *specific* shapes, and also its simplicity and rapidity.

## Results

**Fig. 2:**
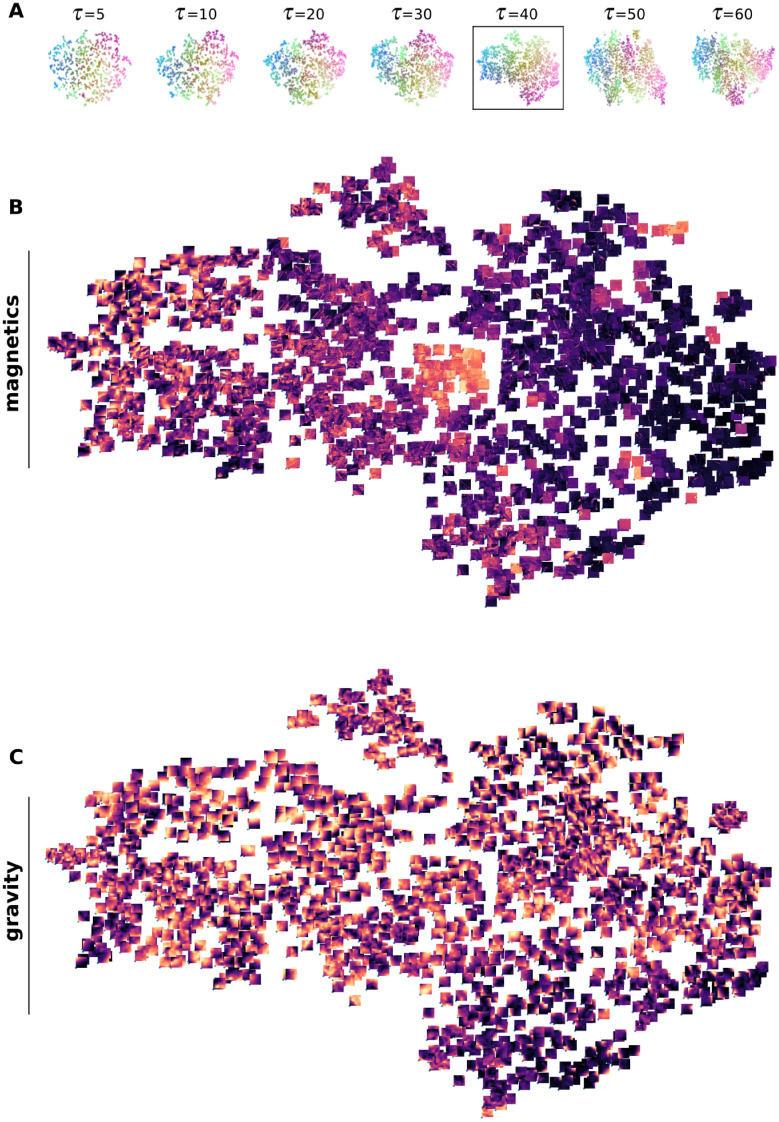
The t-SNE image atlas. (**A**) Perplexity consistency test, with perplexity $\tau =40$ as the reference t-SNE for color mapping. The t-SNE image atlas composed by the individual “maps” of (**B**) magnetic and (**C**) gravity tiles positioned on their respective locations from the reference t-SNE. The interactive plot is available at https://www.loopwms.xyz/wagravmag/.

Firstly, we carry out a perplexity consistency test on a set of embeddings to select one that offers a balanced representation of both global and local structures, and also to ensure that it robustly maintains cluster integrity across different values of the hyperparameter perplexity (see Methods). Figure 2.A shows the results using an embedding with perplexity $\tau =40$ as the reference t-SNE for color mapping the points across all the other embeddings. For the sake of space, results are shown for the range $\tau \in (5, 60)$. Upon comparing the embeddings, one observes that the majority of points with similar colors are still neighbours, therefore demonstrating a general consistency across the range of tested values. This consistency in cluster formation provides confidence in selecting a perplexity value of 40 as an effective representation of the data in a lower-dimensional space, thereby capturing the intrinsic structure of the high-dimensional space effectively.

Each map from the resulting t-SNE atlas is shown in Fig 2.B-C. A visual inspection of this static representation indicates that similar features in geophysics will be observed in the same embedding location, as, for instance, all of the 1663 gravity tiles are spread out in such a way that they form clusters of similar elements in terms of texture, and the same applies to the magnetic data. However, as we will discuss later, a more careful investigation utilizing the interactive tool reveals the existence of a more nuanced structure (see the discussion of Region 2 from Fig. 4) The implications of this arrangement are threefold. Firstly, it demonstrates that the use of texture descriptors for encoding, followed by feature fusion, provides a meaningful and consistent representation of the geophysicalsignatures within the tiles. Secondly, the t-SNE embedding is effective in providing a comprehensible 2D representation of the cluster structure that occurs in the 26D “Haralick-space”. Finally, it also reveals that the dataset itself contains a substantial number of samples (i.e., the tiles) with similar and diverse geophysics, which allows the formation of clusters in the 26D space. This also supports the segmentation strategy applied to the geophysical images as tiles of size $40\times 40$.

**Fig. 3:**
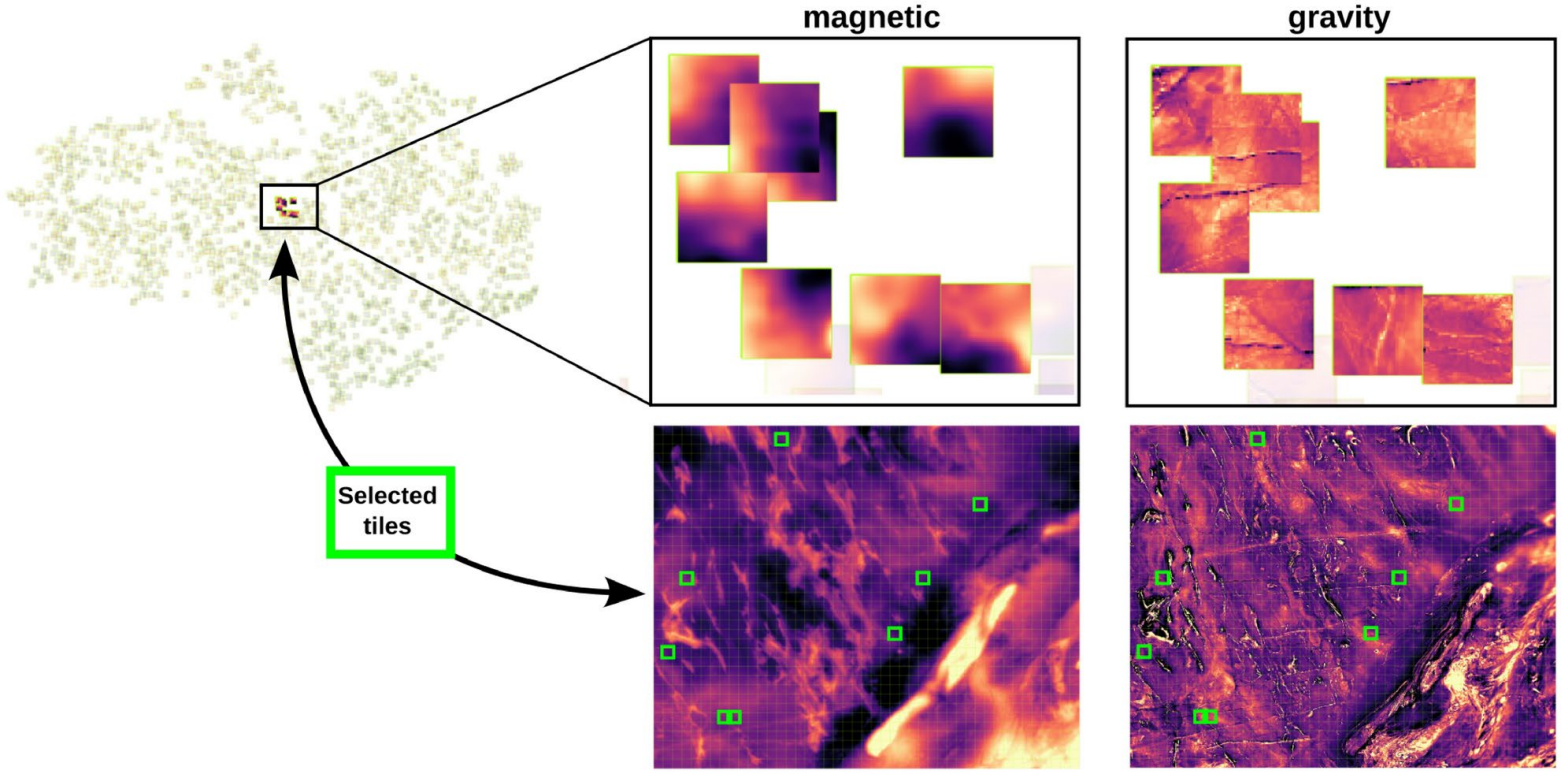
Illustrative depiction of the interactive t-SNE image atlas. Data can be interrogated on both the t-SNE 2D space and the original geographical position. Also, specific tiles from different geophysics can be selected and mapped back to geographical position, and vice-versa.

A deeper analysis can be done by utilizing the interactive visualization tool, available at https://www.loopwms.xyz/wagravmag/. Its capabilities are depicted in Figure 3. It allows the user to explore the t-SNE atlas by moving back and forth between gravity and magnetic data, zooming in to examine different areas, select specific tiles and to map them back to the original geographical location (and vice-versa).

**Fig. 4:**
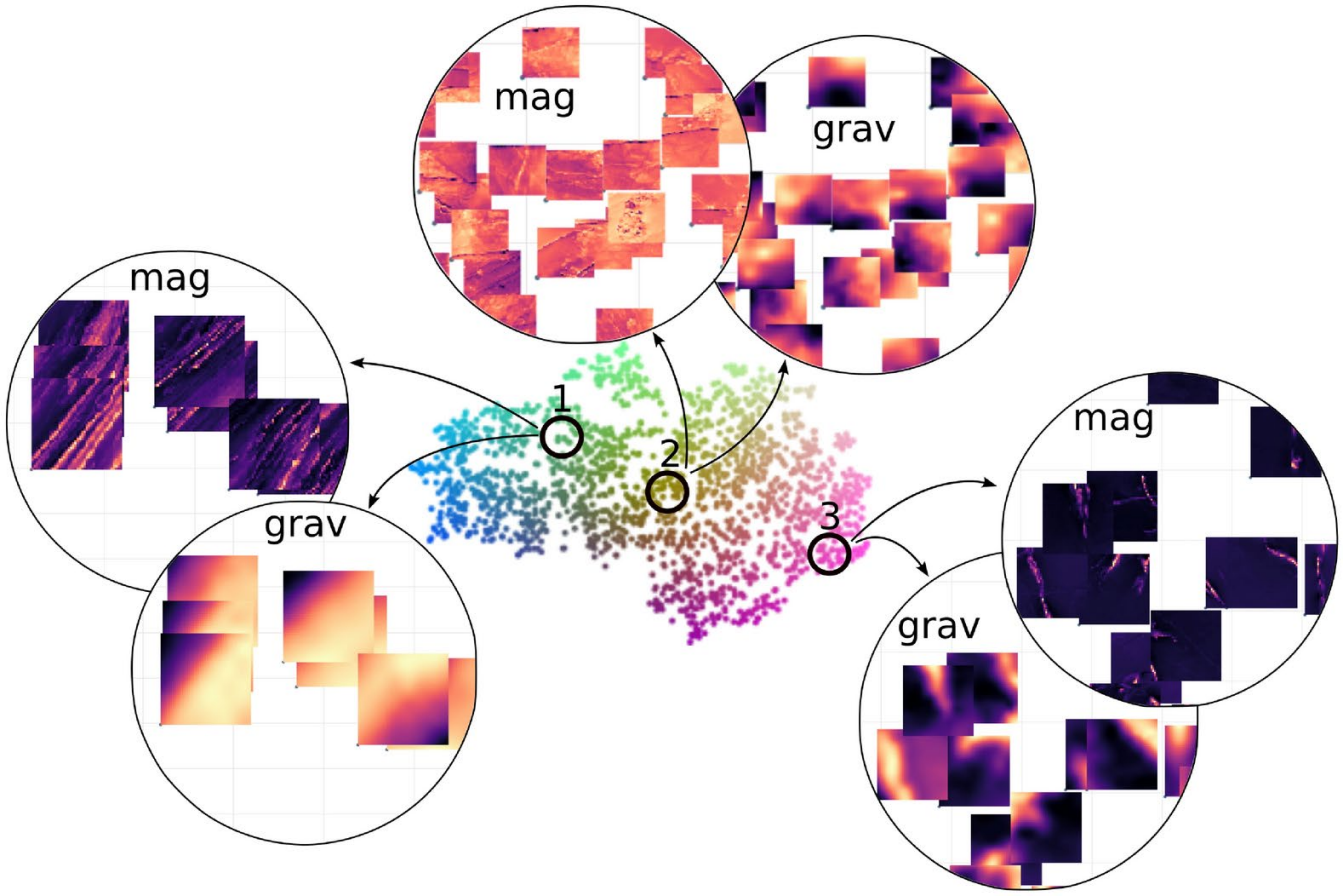
Three regions selected from the t-SNE image atlas containing distinct non-trivial connections between geophysical features.

Therefore, the subject matter expert can utilize the interactive t-SNE atlas as a “navigation map”, facilitating the discovery of new insights, the formulation of hypotheses, and the exploration of non-trivial connections among various geophysical data sets. To demonstrate this, we have highlighted three distinct regions within the t-SNE atlas, as depicted in Fig. 4:

- Region 1 – This region is characterized by a series of tiles with groups of parallel linear magnetic highs accompanied by a very smooth gravity gradient. Moreover, the steepest gravity gradient is perpendicular to the orientation of the linear magnetic highs. These characteristics suggest that these particular tiles could correspond to areas with a dipping stratigraphy.
- Region 2 – A bright orange section in the middle of the magnetic data in t-SNE map (Fig. 2B) immediately draws one attention to this region. It consists of a series of tiles dominated by linear and point magnetic lows, suggesting they are remnant dykes and other intrusions. Interestingly, there is no visually clear pattern in the associated gravity tiles, indicating that this cluster in the data is largely influenced by the magnetic signal with those linear lows.
- Region 3 – In this region the tiles are dominated by isolated linear magnetic highs, which coincide with linear gravity highs. Thus, the geological phenomenon responsible for these features must possess both high density and significant magnetic susceptibility, possibly representing a thicker stratigraphic package. In reality, several factors can cause linear gravity and magnetic highs, but this is where geologists step back into the picture.

**Fig. 5:**
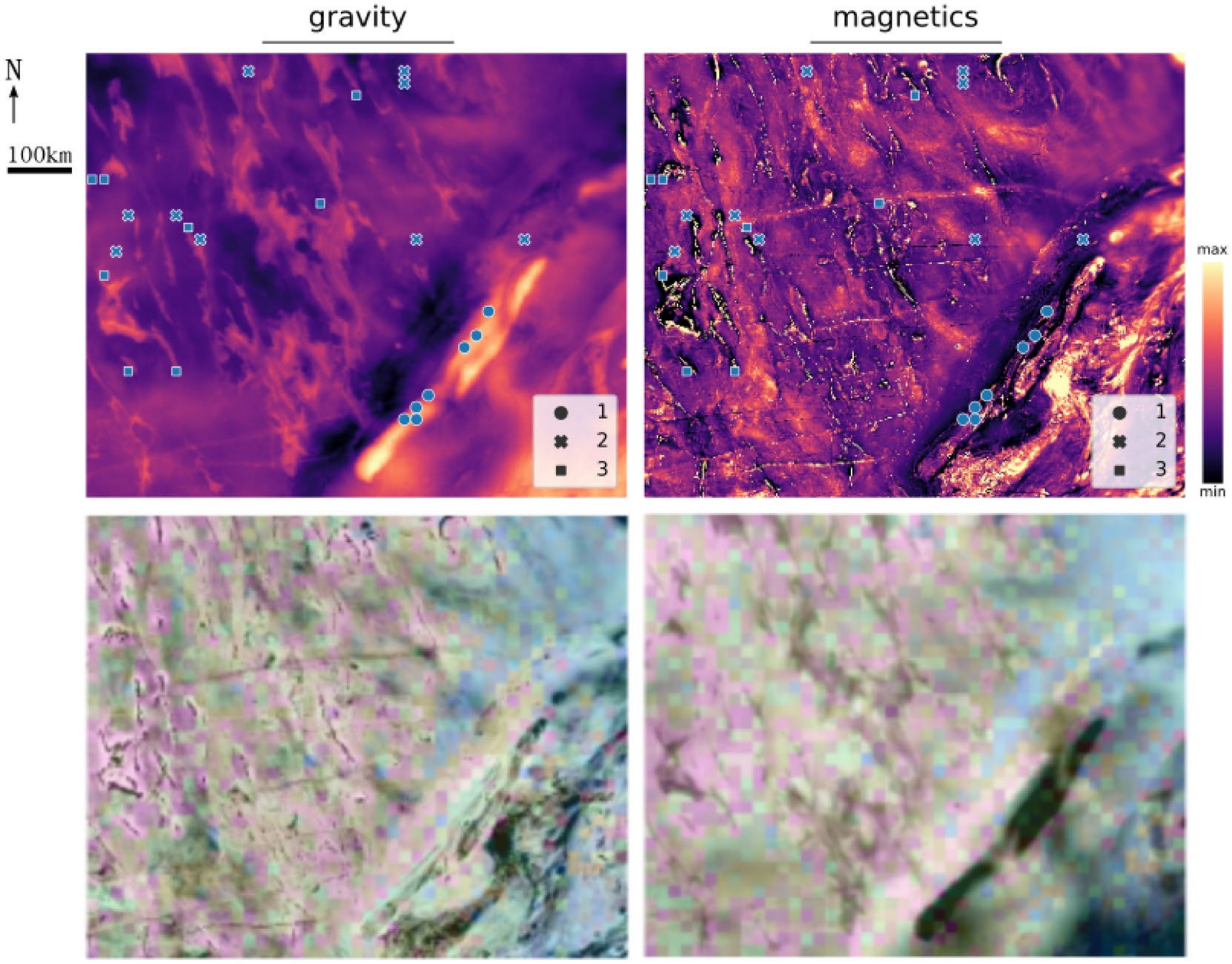
Top panel: samples from the three regions of interest are mapped back to their physical location. Bottom panel shows the image blending of the geophysics and the t-SNE colour from Fig. 4.

For illustrative purposes, Fig. 5 shows the mapping between the image tiles from the t-SNE representation and their original geographical positions. The top panel shows some samples from the 3 aforementioned selected regions. The samples from regions 2 and 3 illustrate that the respective (integrated) geophysical signatures can be found in samples from very different geographical locations, suggesting that the similar underlying geology might be found far apart from each other in the real-world coordinates. Conversely, the (integrated) geophysical features of the samples from region 1 are related to complex sheared boundary of Biranup Zone and Northern Foreland on the western edge of the Fraser Zone within the Albany-Fraser Orogen^35^. Finally, the bottom panel illustrates the overall structure seen in the t-SNE and its relationship with the original geographical positions by *blending* the gray scale image of the geophysical datasets with the the color code scheme from the reference t-SNE (see Methods). This approach not only demonstrates the correspondence between the geophysical data and the abstract clustering in t-SNE but also provides an intuitive visual link between the two, thereby enhancing the interpretability of the spatial patterns observed. Emerging spatial patterns can prompt further investigation to understand the relationship between the underlying geology and geophysical response.

## Discussion

In this study, we present a data-driven method for analyzing gridded geophysical data that allows earth scientists to search for patterns in their datasets without preconceived notions of the specifics of these patterns. It also leverages the integration of diverse gridded datasets, facilitated by the feature fusion of the Haralick texture descriptors, to autonomously reveal intrinsic patterns and significantly enhancing the interpretive landscape for earth scientists. Thus, it allows one to discover * naturally* occurring patterns, as for instance the coincident linear highs, but also highlighting the commonalities in geophysical response of remnant mafic dykes across the case study region. Of course these features can be explored for manually with a geographic information system (GIS); however previous attempts to automate this process often result in too many false positives. This methodology has been demonstrated using magnetic and gravity data from the east Yilgarn Craton region in Western Australia, showcasing its potential in a real-world setting. However, the versatility of our approach allows for its application to any gridded dataset, including gamma-ray spectrometry data (radiometrics), satellite imagery, and digital terrain models. This adaptability opens the door for cross-disciplinary applications, potentially transforming how geoscientists interact with and interpret gridded datasets.

There are many possible extensions to this work. At the present time we only explore a fixed-size of image tiles, but different-sized tiles could enable us to develop a multi-scale Atlas that highlights different relationships at different scales, and ensures that discrete features are captured at the appropriate scale. It should be noticed, however, that the tile scale is crucial because it determines the level of detail captured from the geophysical data. Larger tiles tend to mix multiple geological features, which can dilute the subtle textural variations that Haralick descriptors are designed to quantify. This mixing may lead to less distinct clusters in the t-SNE projection. In contrast, if the tiles are too small, they might not contain enough spatial context to provide robust texture information, resulting in unstable or less meaningful descriptor values. Additionally, overlapping tiles in a sliding window approach could prevent small geophysical anomalies being split across neighbouring tiles, therefore improving the continuity of data interpretation. Our approach is also easily extensible to 3D datasets, and exploratory work is currently being undertaken to help us understand the outputs from stochastic inversion codes for the rapid, streamlined analysis of 100’s of 1000’s of models in a single atlas representative of the search space. Finally, the feature parameterization (Haralick descriptors) and 2D projection (t-SNE) steps could be replaced by other techniques that fulfil similar goals (such as dissimilarity metrics^17^ and Multi-Dimensional Scaling or Uniform Manifold Approximation and Projection, respectively). An interesting option for the featureparametrization is the use of the output from the intermediary layers of a convolutional neural network^36^.

The strength of the workflow presented here is that it is not based on prior assumptions of feature geometry and and integrates information from diverse gridded datasets, and thus can be used to navigate single band or multi-band Earth Science data in a data-centric fashion, which allows one to discover patterns in grid data that may be missed by more conventional techniques. Specifically, our approach is fundamentally data-driven because it abstains from predefined geological models or feature assumptions in favor of letting the data reveal its inherent structure. By fusing Haralick texture descriptors into a unified high-dimensional feature space, an unbiased representation of the data is achieved, while the subsequent t-SNE dimensionality reduction highlights natural clusters and relationships without imposing any prior interpretive framework. Consequently, the resulting visualization – the atlas – is a direct reflection of the underlying data patterns and facilitates exploratory analysis that can be readily extended to additional gridded datasets.

Our study offers practical applications that extend beyond simple clustering analysis by providing valuable insights to geologists. For instance, our atlas can help exploration geologists identify specific geological settings and then assess the mineral prospectivity of focused areas. By quantifying and visualizing subtle geophysical patterns, our tool provides a data-driven framework that can highlight areas of interest for focused exploration. Additionally, the atlas can serve as a valuable pre-planning resource for exploration projects, enabling geologists to pinpoint promising locations to start their exploration journey which could improve their discovery timeline.

## Conclusion

In this work, we introduced a novel, data-driven methodology for the integrated interpretation of geophysical datasets. By encoding data patches from both magnetic and gravity images using Haralick texture descriptors, we fused these complementary datasets into a unified 26-dimensional feature space. This integration allows the t-SNE algorithm to simultaneously embed and visualize the combined geophysical signatures, thereby letting the inherent structure of the data to emerge and enhancing the resolution of subtle geological features.

When applied to the case study area, our approach successfully generates an interactive t-SNE atlas that not only reveals distinct clusters corresponding to various geophysical patterns but also directly links these clusters back to their original geographic locations. This capability enables a more intuitive and comprehensive exploration of complex geological structures, supporting both hypothesis generation and more targeted mineral exploration. To illustrate this, our analysis identified three distinct geophysical regions in the t-SNE atlas that underscore the capability of our methodology to reveal complex geological patterns. The first region features clusters of tiles with parallel linear magnetic highs and a smooth, perpendicularly oriented gravity gradient, suggesting dipping stratigraphy. The second region is characterized by a cluster dominated by linear and point magnetic lows – indicative of remnant dykes and intrusions-with little corresponding gravity signal. This exemplifies a case where the subsurface configuration highlights the predominance of magnetic data for inferring its properties. Finally, the third region exhibits isolated linear magnetic highs that align with linear gravity highs, suggesting a geological phenomenon with high density and magnetic susceptibility, possibly a thicker stratigraphic unit.

Overall, our methodology demonstrates significant added value by automating the identification of geological patterns, and enabling a more objective, data-centric analysis of geophysical datasets. The unified feature space effectively integrates the information content from the magnetic and gravity datasets, improving the robustness of geophysical interpretation compared to analyzing each dataset in isolation. It should be noticed that our method was *demonstrated* using magnetic and gravity data. However, encoding information through Haralick texture descriptors allows for data-fusion capability that makes it versatile and readily extendable to other gridded datasets, such as gamma-ray spectrometry, satellite imagery, and digital terrain models. Consequently, we anticipate future applications incorporating a broader variety of data types. Future work will focus on extending this framework to additional data types and scales, further enhancing its applicability across various geological settings.

## Methods

### Geological setting and data set

The Palaeo-Neoarchean Yilgarn Craton in Western Australia has a long-lived and complex tectonic history and is subdivided into seven terranes^37,38^.The relevant part of our study is the Eastern Goldfields Superterrane, which hosts substantial NNW-trending greenstone belts separated by granite and granitic gneiss. The greenstone belts are conspicuous in geophysical data, being strong positive magnetic and gravity anomalies. The southeastern part of our study area is part of the Albany-Fraser Orogen which is separated into the Biranup, Fraser and Tropicana zones of the Kepa Kurl Booya Province^39^ (Fig. 2). The 1815-1625 Ma Biranup Zone is dominated by strongly deformed orthogneiss and minor metagabbro and hybrid rocks^39^. The Fraser Zone is conspicuous in potential field data with an anomalously high gravity and magnetic signature. The high-density rocks respond to the widespread occurrence of metagabbroic rocks^40^.

The geological setting is a large area from the east Yilgarn Craton region in Western Australia (WA), comprising a rectangular field with east-west (EW) dimension of $840~\textrm{km}$ and the north-south (NS) dimension of $700~\textrm{km}$. To illustrate the method and its capabilities in integrating different types of gridded data, we selected the corresponding magnetic and gravity anomalies of this area^32,33^. Figure 1.A shows the geophysical data overlaid on the geological map of WA. Each geophysical image is represented by a $1826 \times 1512$ grid, with each grid cell corresponding to an area of $450~\textrm{m} \times 450~\textrm{m}$, with the magnetic data being upsampled to match the gravity data spatial resolution.

### Tiling and image enhancement

This section describes the cropping and segmentation of the original magnetic and gravity images into small tiles and the subsequent re-scaling of the tiles grey scale levels.

Firstly, the tiles size for the segmentation of the grid must be selected. The guiding principle is that the t-SNE will group tiles with similar features in geophysics. If the tile size is too large, one incur on the risk of having too many different and complex geophysical signatures within each tile, and specific local nuances will be lost. If the size is too small, there will be a lack of information content to differentiate the tiles, or the lack of an actual signature that can be associated to geophysics. After careful visual inspection, we opted for square tiles of size $n\times n$, with $n=40$ pixels, which provided a good balance between both scenarios. This corresponds to a physical area of dimensions $18~\textrm{km} \times 18~\textrm{km}$. In mineral exploration terms, this can be considered to be from camp to prospect scale, at a scale where important exploration decision need to be made in terms of the targeting of favourable areas. In order to have an integer number of tiles, each image was cropped (in the east and in the north; see Supplementary Fig. 1 middle panel) resulting in a $1800 \times 1480$ grid, corresponding to ($720~\textrm{km} \times 592~\textrm{km}$). This resulted in $N=1665$ tiles for each geophysical dataset. Due to the large difference in pixel intensity distribution, some tiles appear almost flat (i.e., homogeneous; see Supplementary Fig. 1). To enhance the informationcontent, *each* tile underwent amplitude normalization by re-scaling the pixel intensities to (0, 255). Figure 1.A illustrates the process by showing the cropped images and the relative size of one tile indicated as a black square. All tiles are shown in the Supplementary Fig. 1 bottom panel.

In summary, the working data comprises a total of 3330 image tiles, grouped in two sets with 1665 images from each geophysical dataset: one for magnetic data and the other for gravity data. Conceptually, the elements of each geophysical set are represented as points in two very high-dimensional spaces with dimension $n^2=1600$.

### Texture descriptors

In this section, our goal is to obtain a low *m*-dimensional representation of the image tiles from each geophysical dataset, such that $m<< n^2$ while capturing the essential features of the geophysical datasets. For that, we will make use of the Haralick texture descriptors, a now classic technique in digital image processing.

In a series of papers, Haralick and co-authors summarized and expanded the statistical foundations of texture analysis based on the concept of the grey level co-occurrence matrix (GLCM)^26,27^ – under this framework the intensity of a pixel in a image is referred as the pixel’s grey level. The GLCM captures the information of the difference in grey level of two neighbour pixels given a distance *d* and an angle $\theta$ between them.

**Fig. 6:**
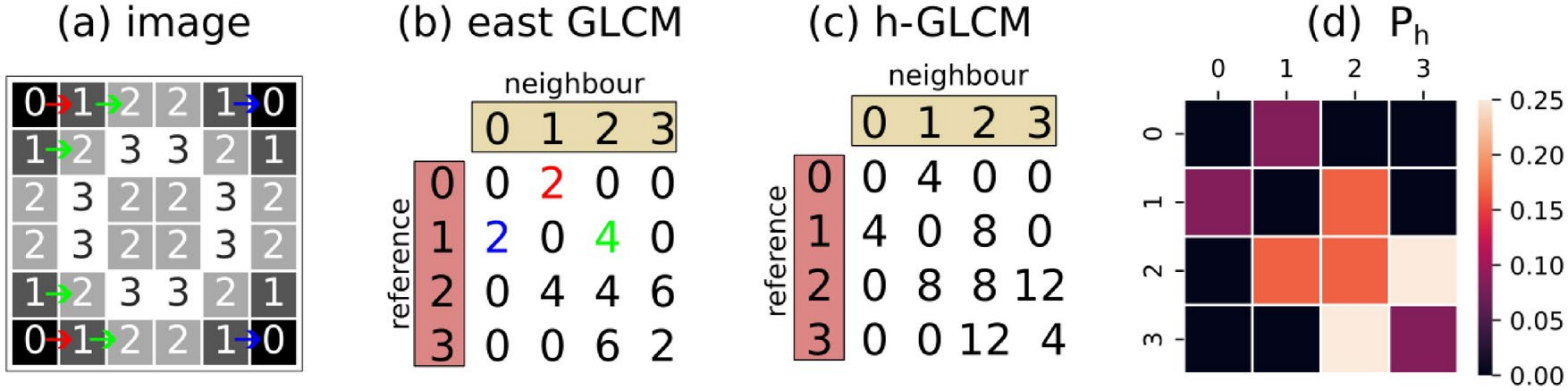
Illustrative example of the calculation of a GLCM with $(d, \theta ) = (1, 0^{\circ })$.

Figure 6 shows the steps to calculate the GLCM for $(d, \theta ) = (1, 0^{\circ })$ from an illustrative image of size $6 \times 6$ in a four-level quantization ($n_g = 4$, ranging from 0 to 3).

Firstly, the east (right) GLCM $A \in \mathbb {Z}^{n_g \times n_g}$ is calculated by counting the number of co-occurrences of all combinations of grey levels between a *reference* pixel and its *neighbour* (Fig. 6b). For instance, there are two occurrences of a reference pixel with grey level 0 and a (right) neighbour with grey level 1 (highlighted in red). Secondly, an horizontal GLCM (h-GLCM) $A_{0^{\circ }}$, encoding horizontal structure information (i.e., $\theta = 0^{\circ }$), is calculated as $A_{0^{\circ }} = A + A^{\top }$. This step is equivalent to adding the east and west GLCMs, without necessity for calculating the latter. Finally, the h-GLCM is normalized by the sum of the elements $a_{ij}$ of $A_{0^{\circ }}$ as

1 $$\begin{aligned} P_{0^{\circ }} = \frac{A_{0^{\circ }}}{\sum _{i,j=1}^{n_g}(a_{ij})}. \end{aligned}$$

Therefore, the normalized GLCM $P_{0^{\circ }} \in \mathbb {R}^{n_g \times n_g}$, with elements $p_{i,j} \in [0,1]$, is the empirical estimate of the joint probability distribution of two (*d*-neighbours) pixels having grey levels differing by $k = i-j$, with $k \in \{0, 1, ..., n_g-1\}$. As it is standard in the literature, for now on we refer to the normalized GLCM simply by GLCM.

The *texture descriptors* are ways of summarizing different aspects of the GLCM. For instance, the 9th descriptor utilizes the concept of entropy ^41^ to express the complexity of tonal co-occurrences in the image as

2 $$\begin{aligned} f_9 = -\sum _{i,j=1}^{n_g} p_{ij}\log (p_{ij}). \end{aligned}$$

Each descriptor is calculated four times from different GLCMs covering the directions $\theta \in \{0^{\circ }, 45^{\circ }, 90^{\circ }, 135^{\circ }\}$. Finally, the average of these values is taken, resulting in a descriptor that is approximately invariant to rotations. Hereafter, the term “descriptor” will refer to this average value. Haralick suggested a total of 14 descriptors: contrast, correlation, sum of squares variance, inverse difference moment, sum average, sum variance, sum entropy, entropy, difference variance, difference entropy, information measure of correlation 1 and 2, and maximal correlation coefficient. The 14th descriptor is known to be computationally unstable^42,43^, and it is often not integrated in the analysis. The reader is referred to Supplementary Table 1 for the list of descriptors and their mathematical definitions. For an in-depth and accessible discussion about the Haralick descriptors and best practices, we refer the reader to Refs.^24,25^.

Here, we utilize the first 13 original Haralick texture descriptors to encode the image tiles as points in a 13-dimensional space as follows. Firstly, the descriptors are calculated for each image tile with the Python package for computer vision Mahotas^42^. For magnetic data, the $i^{th}$ image tile is then represented by the point ${\textbf {f}}_i^{(m)} = (f_{i,1}^{(m)}, ..., f_{i,13}^{(m)}) \in \mathbb {R}^{13}$, and the $i^{th}$ gravity image tile by ${\textbf {f}}_i^{(g)} = (f_{i,1}^{(g)}, ..., f_{i,13}^{(g)}) \in \mathbb {R}^{13}$. The input data can be represented as $n \times M$ matrices, with $n=1665$ samples and $M=13$ descriptors

In matrix form, the whole datasets can be represented as $F^{(m)} = [f^{(m)}_{i,j}]$ and $F^{(g)} =[f^{(g)}_{i,j}]$, with $i=1, ..., 1665$ and $j=1, ..., 13$, with the rows corresponding to the samples (i.e., tiles) and columns corresponding to the 13 texture descriptors. Finally, each column of both matrices is standardized by subtracting its mean and dividing by its standard deviation. We will call the standardized matrices $\widetilde{F}^{(m)} = [\widetilde{f}^{(m)}_{i,j}]$ and $\widetilde{F}^{(g)} =[\widetilde{f}^{(g)}_{i,j}]$. To keep notation simple, we will refer to the respective standardized data points simply as ${\textbf {f}}_i^{(m)}$ and ${\textbf {f}}_i^{(g)}$.

### Feature fusion

The texture descriptors representation of the image tiles from magnetic data, ${\textbf {f}}_i^{(m)}$, and gravity, ${\textbf {f}}_i^{(g)}$, provides a straightforward way to combine the different types of geophysical data. Conceptually, this is achieved through the direct product $\mathbb {R}^{13}\times \mathbb {R}^{13}$ of the individual 13-dimensional feature spaces of the magnetic and gravity datasets. Specifically, we define the fused feature vector as ${\textbf {f}}_i^{(m, g)} \doteq (f_{i,1}^{(m)}, ..., f_{i,13}^{(m)}, f_{i,1}^{(g)}, ..., f_{i,13}^{(g)}) \in \mathbb {R}^{26}$ as a representation of the joint magnetic and gravity characteristics of the $i^{th}$ geographical location. The combined dataset $\widetilde{F}^{(m,g)} = (\widetilde{F}^{(m)}, \widetilde{F}^{(g)}) \in \mathbb {R}^{1665,26}$ is then constructed by horizontally stacking the standardized feature matrices from the two data types.

It is important to note that we employ the concept of feature fusion by concatenating the feature vectors derived from the respective GLCM matrices. We are not directly multiplying the GLCM matrices; rather, we perform a direct product of the two 13-dimensional feature spaces. This fusion preserves the independent characteristics of both magnetic and gravity data while enabling an integrated analysis of the combined features.

### t-SNE projection

We start by providing an overview of the t-SNE algorithm, followed by a description of its application in our workflow.

The t-Distributed Stochastic Neighbor Embedding^31^ (t-SNE) is a nonlinear embedding technique to visualizing high-dimensional data in lower dimensional space (usually 2D or 3D), thereby thicker stratigraphic ualing underlying patterns and structures that might be otherwise obscured. It models pairwise similarities between data points ${\textbf {x}}_i\in \mathbb {R}^{r}$, from the original high r-dimensional space, to the points ${\textbf {y}}_i\in \mathbb {R}^{s}$ in the lower s-dimensional embedding, and then iteratively optimizes the latter to best represent the former. Unlike traditional linear techniques, t-SNE focuses on preserving the local and global relationships between data points. The balance between these opposing aspects, or the emphasis in one of them, is regulated by the user-defined hyperparameter perplexity, $\tau$ (explained in more details later). This flexibility makes the t-SNE particularly effective at capturing complex nonlinear relationships. It has found applications in diverse domains, such as biology^44^, natural language processing^45^, geochemistry^20^, domain interpretation in geological data^23^, and has been extensively utilized to visualize cellular heterogeneity in the context of single-cell RNA sequencing^46^. For best practices, pitfalls and advanced usage, we refer the reader to Refs.^28–30^.

A central idea of t-SNE is defining the pairwise similarities of data points through probabilities instead of (metric) distances. In brief, the algorithm works as follows. In the *original* high dimensional space, a distance (metric) function $\left\| \bullet \right\|$ between points ${\textbf {x}}_j$ and ${\textbf {x}}_i$ is mapped to the conditional probability of picking ${\textbf {x}}_j$ as a neighbour of ${\textbf {x}}_i$ under a Gaussian distribution $\mathscr {N}({\textbf {x}}_i,\,\sigma _i^{2})$:

3 $$\begin{aligned} p_{j \mid i}=\frac{\exp \left( -\Vert {\textbf {x}}_i-{\textbf {x}}_j\Vert ^2 / 2 \sigma _i^2\right) }{\sum _{k \ne i} \exp \left( -\Vert {\textbf {x}}_i-{\textbf {x}}_k\Vert ^2 / 2 \sigma _i^2\right) }. \end{aligned}$$

Then, the similarity between those points is taken as the joint probability $p_{ij}=\frac{1}{2}(p_{j \mid i} +p_{i \mid j})$. In the original paper^31^, the distance function is the Euclidean distance. However, other distance metrics can be utilized, and several are implemented in software packages. For instance, the cosine distance is often suggested when dealing with very high dimensional space due to the curse of dimensionality, and the Aitchison distance has been suggested in the context of geochemical assays^20^. The width of the Gaussian distribution, $\sigma$, is related to the user-defined hyperparameter *perplexity*, $\tau$. It controls the weight the distance between neighbours has in calculating their similarity: the larger the $\sigma$, the farther the neighbors that are effectively taken as similar. Consequently, the perplexity $\tau$ controls the balance between capturing the local and global aspects of the data, influencing the quality and interpretability of the resulting t-SNE. Larger (smaller) values of $\tau$ puts more weight on preserving the global (local) structure of the data. Intuitively, one can think of $\tau$ as related to the number of nearest neighbors that is considered around a particular point.

On the *lower dimensional embedding*, the similarity between points ${\textbf {y}}_j$ and ${\textbf {y}}_i$ is modeled as a Student t-distribution with one degree of freedom:

4 $$\begin{aligned} q_{ij}=\frac{\left( 1+\Vert {\textbf {y}}_i-{\textbf {y}}_j\Vert ^2\right) ^{-1}}{\sum _{k \ne l}\left( 1+\Vert {\textbf {y}}_k-{\textbf {y}}_l\Vert ^2\right) ^{-1}} \end{aligned}$$

where the distance function $\left\| \bullet \right\|$ is the euclidean distance. Finally, the t-SNE algorithm iteratively rearranges all points ${\textbf {y}}_{{\textbf {j}}}$ using a gradient descent method to minimize the Kullback-Leibler divergence between $p_{ij}$ and $q_{ij}$.

Here we apply the t-SNE to the data matrix $\widetilde{F}^{(m,g)}$ using the Python package openTSNE^47^. This package implements the best practices as described in Ref.^28^, based on previous ideas in the literature^48–50^. Namely, PCA initialization, increased learning rate and multi-scale similarities – the latter relevant for large datasets (e.g., $N>>100,000$).

From the several hyperparameters to tune, the most important is the perplexity $\tau$. Originally^31^, it was suggested that the t-SNE is fairly robust regarding changes in $\tau$, with typical values between 5 and 50. Practice had supported the idea that $\tau \approx 30$ offers an optimal balance between preserving the global and local structures. Nevertheless, it is common to find in the literature perplexity values ranging from 30 to 80. More recently, a few methodologies^28,51^ have been proposed for selecting the perplexity value in terms of the sample size, for instance $\tau =10N/512$ or $\tau =N/100$ – in our case $\tau =32$ and $\tau =17$, respectively. For very large datasets (e.g., $N>>100,000$), it has been suggest a multi-scale approach, using first a large $\tau$ followed by a smaller one. However, the influence of $\tau$ on the t-SNE is more nuanced, and depends not only on the dataset size, but on its density, clustering structure and what aspect of the data the data analyst wants to highlight. Reference^30^ recommends that “*getting the most from t-SNE may mean analyzing multiple plots with different perplexities*”.

Considering those different ideas, we established a *perplexity consistency* test (see Fig. 1B, middle panel). The primary objective of it is to examine the consistency of the resultant low-dimensional embeddings in terms of clustering structure. Firstly, the t-SNE is applied to the data matrix for various perplexity values around the prescribed candidates. In our case it implies in $\tau \in (5, 10, 20, 30, 40, 50, 60, 70, 80)$. Secondly, we select the t-SNE with an intermediary perplexity value (e.g., $\tau =40$) as the reference, and associate each point *i* to an RGB color by using its coordinates and distance from the center. For instance, if the coordinates of point *i* are ${\textbf {y}}_{{\textbf {j}}}=(y_{i1}, y_{i2})$, then its RGB color is $\textrm{R}_{\textrm{i}}={\textrm{y}}_{{\textrm{i1}}}$, ${\textrm{G}}_{\textrm{i}}={\textrm{y}}_{{\textrm{i2}}}$ and ${\textrm{B}}_{\textrm{i}}=\sqrt{{\textrm{y}}_{{\textrm{i1}}}^2+{\textrm{y}}_{{\textrm{i2}}}^2}$. The color codes from the reference plot are then used to color the points in all the different t-SNE plots, allowing one to assess through visual inspection whether similar clusters are preserved across different perplexity settings. If the Haralick texture encoding captures the geophysical nuances of the dataset, and also if the dataset itself contains samples with similar and diverse geophysics allowing the formation of clusters in the 26D space, then we expect to observe not only a clear clustering structure but also a general consistency of it in the range of tested values around the reference t-SNE. In that case, we select the reference t-SNE to build the t-SNE image atlas.

### Image atlas

We use the reference t-SNE to build a set of two visualizations, which we together call the *t-SNE image atlas* (see Fig. 1B, bottom). This is done by positioning the tiles from magnetic and gravity data according to the reference t-SNE coordinates $(y_{i1}, y_{i2})$, using the Python package Bokeh^52^. In this process, one may use either the original or the re-scaled tiles within the Atlas. For the current dataset, we found that using the latter greatly enhances the perception of nuances in geophysics of those images, thereby facilitating the geoscientific analysis.

Our proposal is to use the Atlas as a “navigation map”, serving as a tool to uncover new insights, formulate hypotheses and search for non-trivial connections across different geophysical datasets. Positioning the tiles using the t-SNE coordinates allows one to interrogate the data by its similarities in geophysical response, independent of the tiles’ actual geographical locations. The value of this approach is further augmented by interactive features such as zooming and panning, transforming the Atlas into a dynamic tool for geophysical exploration.

### Image blending

It could be beneficial to inspect how the overall cluster distribution of tiles in the t-SNE is preserved in the original geographical location. For instance, are they still clustered on the original geographical location, or are they spread in very different places? Emerging spatial patterns can prompt further investigation to understand the relationship between the underlying geology and geophysical response.

**Fig. 7:**
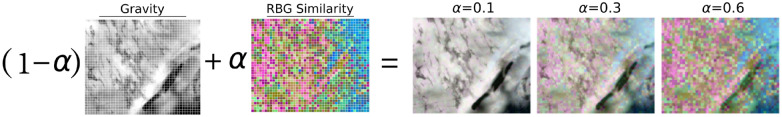
Illustrative example of the image blending between the image representation of the gravity dataset and its correspondence to the abstract clustering in t-SNE.

For that, we want to have a visualization that brings together a given geophysical dataset (e.g., gravity) and the color code from the points of the reference t-SNE plot. Because each tile of the geophysical dataset corresponds to a specific point in the t-SNE, using its color code is straightforward. The overall idea is to use the Python package Pillow^53^ to blend two RGB images. The blending process is shown in Fig.7. We will exemplify the process using the gravity data.

This is implemented by firstly generating an RGB representation of the gray scale image of the gravity data. By plotting this data in gray scale and using the method *tostring_rgb()* from Matplotlib^54^, we generate three matrices representing the RGB channels. Secondly, we create an empty grid of size $1800 \times 1480$, corresponding to the size of the gravity dataset and applying the same segmentation process to generate 1600 tiles of size $40\times 40$ pixels. Using the known correspondence of those empty tiles to the RGB color-coded points from reference t-SNE plot, we apply the values of the red channel R to those tiles. Then, the segmentation process is reversed by blocking thetiles together into a single $1800 \times 1480$ grid. The process is repeated to generated other two $1800 \times 1480$ grids to represent the green and blue values. These three grids (or matrices) are the RGB representation of the similarities in the (fused) Haralick space as expressed in the reference t-SNE plot. The corresponding images of the gray scale plot of the gravity data and the RGB similarity representation are shown on the left of Fig.7. Finally, we use the Pillow function *blend* to generate a new image by interpolating between the two RGB input images, with a parameter $\alpha$ that controls the strength between those images. Low values of $\alpha$ favors the first image, while larger values of favors the second image. Figure7 shows the blending for three values of $\alpha$.

## Supplementary Information

Supplementary Information.

## Data Availability

The magnetic and gravity dataset and source codes are available for downloading at the link: https://github.com/ll-portes/t-sne_atlas. The interactive version of the t-SNE atlas in Fig. 2 is available at https://www.loopwms.xyz/wagravmag/, which was created using the Python package Bokeh ^52^ available at https://bokeh.org/. The Haralick texture descriptors were calculated using the Python package for computer vision Mahotas ^42^ available at https://github.com/luispedro/mahotas. The t-SNE embeddings were performed with the Python package openTSNE ^47^, available at https://github.com/pavlin-policar/openTSNE. Matplotlib ^54^ was used to generate most of the figures in this manuscript and to extract the RGB channels for the image blending process. It is available at https://matplotlib.org/. The Python package Pillow ^53^ was used in the image blending process, and it available at https://python-pillow.github.io/.
