# Supplementary material for: Feature fusion-enhanced t-SNE image atlas for geophysical features discovery
Source: Sci Rep. 2025 May 17;15:17152. doi: 10.1038/s41598-025-01333-3 (PMC12085580; doi:10.1038/s41598-025-01333-3)
Supplement: Supplementary file 1 — Supplementary Information. [file 41598_2025_1333_MOESM1_ESM.pdf]

Feature fusion-enhanced t-SNE image atlas for geophysical discovery. Supplementary Information

Leonardo Portes, Guillaume Pirot, Michel M. Nzikou, Jeremie Giraud, Mark Lindsay, Mark Jessell, and Edward Cripps

August 7, 2024

1 Supplementary Figures

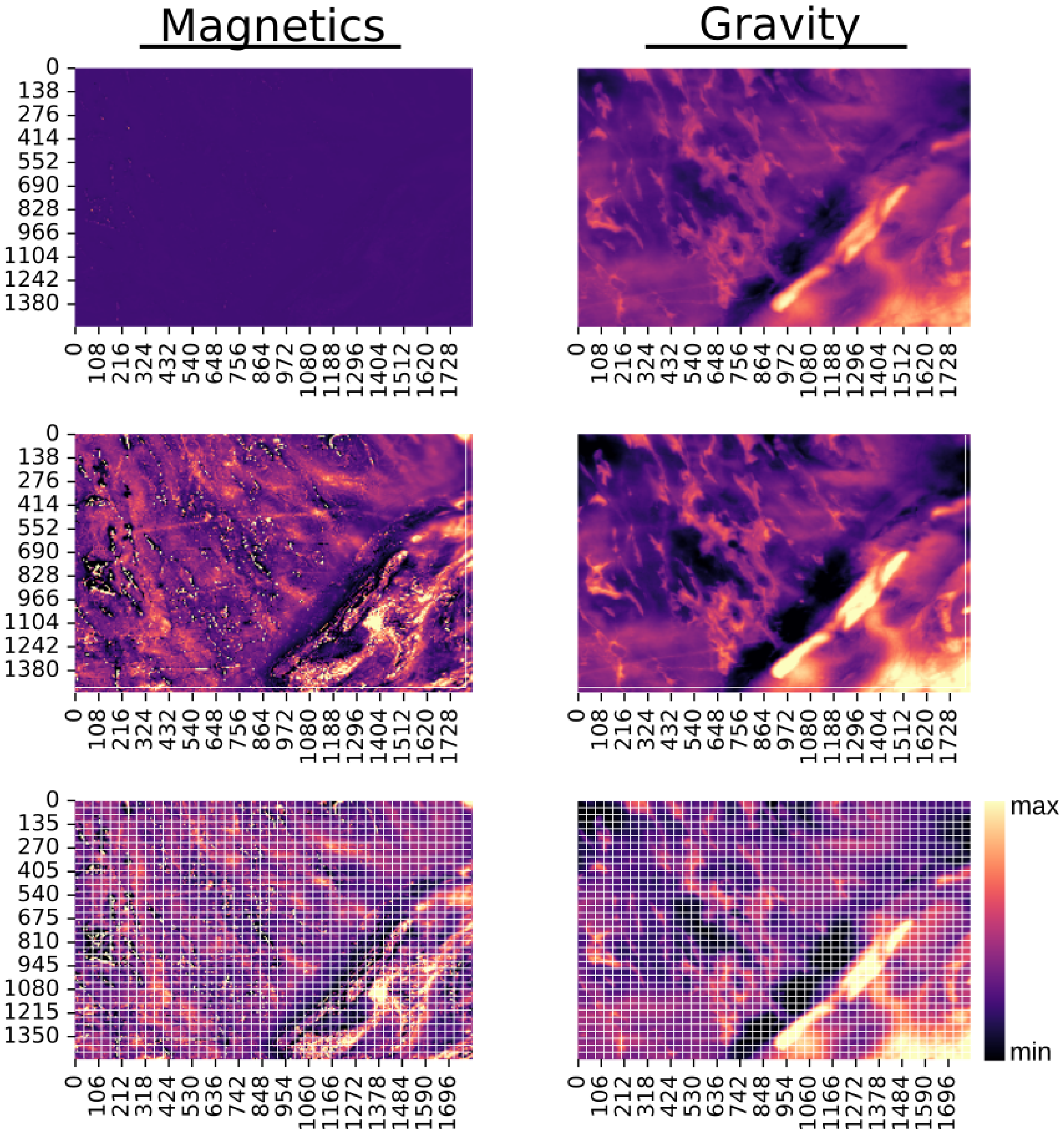

Supplementary Figure 1: Top panel shows the original geophysical responses. The same data is shown in the middle panel with robust quantile colormaping. The vertical and horizontal white lines indicate the cropping limits. Bottom panel shows the cropped images segmented in 1665 tiles of size 40×40 pixels.

2 Supplementary Table

Haralick Feature	Mathematical Expression	Notation
ASM	$\sum_{i,j=1}^{N} P(i,j)^2$	f_1
Contrast	$\sum_{i,j=1}^{N}(i-j)^2 P(i,j)$	f_2
Correlation	$\frac{\sum_{i,j=1}^{N}(i-\mu_X)(j-\mu_Y)P(i,j)}{\sigma_X \sigma_Y}$	f_3
Sum of Squares Variance	$\sum_{i,j=1}^{N}(i-\mu)^2 P(i,j)$	f_4
IDM	$\sum_{i,j=1}^{N} \frac{1}{1+(i-j)^2} P(i,j)$	f_5
Sum Average	$\sum_{k=2}^{2N} k P_{x+y}(k)$	f_6
Sum Variance	$\sum_{k=2}^{2N}(k - \text{Sum Average})^2 P_{x+y}(k)$	f_7
Sum Entropy	$-\sum_{k=2}^{2N} P_{x+y}(k) \log(P_{x+y}(k))$	f_8
Entropy	$-\sum_{i,j=1}^{N} P(i,j) \log(P(i,j))$	f_9
Difference Variance	$\sum_{k=0}^{N-1} k^2 P_{x-y}(k)$	f_{10}
Difference Entropy	$-\sum_{k=0}^{N-1} P_{x-y}(k) \log(P_{x-y}(k))$	f_{11}
IMC_1	$\frac{HXY-HXY1}{\max(HX,HY)}$	f_{12}
IMC_2	$\sqrt{1-\exp(-2(HXY2-HXY))}$	f_{13}

Table 1: First 13 Haralick features and corresponding mathematical expressions. The acronyms are Angular Second Moment (ASM), Inverse Difference Moment (IDM), and Information Measure of Correlation (IMC). Some features are known by different names and expressed in different mathematical notations.
